# News and Notes

**Published:** 1901-07

**Authors:** 


### *NEWS AND NOTES.*

---

THE *Atlanta Medical and Surgical Journal* and *Southern Medical Record* having been consolidated into the ATLANTA JOURNAL-RECORD OF MEDICINE, exchanges will please be sent only to the latter. We are getting many duplicates.

---

DR. H. F. HARRIS is spending the summer in Europe.

---

DR. T. H. HANCOCK is spending some time in Virginia.

---

DR. N. SENN starts on July 4 for a trip around the world.

---

THE use of absinthe in France is said to be on the decline.

---

THE Atlanta Society of Medicine has adjourned for the summer.

---

HAVANA, Cuba, is reported to be entirely free from yellow fever.

---

DR. W. H. HUDSON, of Lafayette, Ala., recently paid THE JOURNAL-RECORD a visit.

---

WITHIN the last year 8,330 persons were arrested on the streets of St. Petersburg for drunkenness.

---

DR. M. B. HUTCHINS presented before the Atlanta Society of Medicine a case of epithelioma of the face healed by the continued use of the X-ray.

THE health officer of Atlanta will be elected by the city council in July. The candidates are Drs. J. P. Kennedy, C. A. Smith, R. W. Westmoreland, and T. H. Kenan.

---

A FORMER professor of the Warsaw University was sentenced to 2½ years in the workhouse, followed by 4 years of police supervision and deprivation of all rights and privileges for having committed rape.

---

DR. L. B. GRANDY, who, for the past two years, has been in the Philippines, has returned to this country, and is now stationed at Presidio, Cal. Dr. Grandy spent some weeks in Atlanta. He expects to return to Manila during the summer.

---

DR. S. E. BRYAN, claiming to be a graduate from an Eclectic school, is suing the Atlanta Society of Medicine for alleged defamation of character. His name was presented to the grand jury along with others for illegal practice of medicine.

---

IN one of the city hospitals for the insane in St. Petersburg a stage was erected on which light vaudevilles are produced. The actors, as well as the audience, are the patients themselves, the former proving in every respect equal to the task, while the latter seem to derive real enjoyment from the affair.

---

THE census shows, says *American Medicine,* that there is one registered physician to every 655 people in the United States, while in Germany, in 1898, there was but one doctor to each 2,114 of population. Some States, however, have proportionately more practitioners than others. In California, for instance, the ratio is as 1 to 416, while in North Carolina it is as 1 to 1,189. This disparity is particularly pointed sometimes in States similarly situated—Michigan's ratio is as 1 to 570, Wisconsin's proportion but 1 to 936, and Minnesota has but one doctor to each 1,004 people.

---

THE London *Lancet* of recent date calls attention to the dangerous ingredient of finely crushed glass splinters added to some high-grade candy to give it a sparkling appearance, and supposed to be adopted to compensate for the absence of any crystalline appearance in glucose, which is used so largely as a substitute for cane-sugar in confectionery. When the candy is dissolved in warm water the splinters fall to the bottom of the tumbler in a tiny heap of broken glass. Two children who ate of this candy shortly afterwards suffered from severe abdominal pain, in one case in the region of the appendix. The pain persisted for several days.

---

Green is the flowery tamarack;
  The aspen's ghost leaves quiver;
In the right hypochondriac
  Still sleeps the torpid liver.

The wind is light, the world is young;
  High leaps the rutting goat;
Upon the back part of the tongue,
  Appears a furry coat.

Beneath the ground are bursting seed:
  In trees the birds are billin';
While men and women seem to need
  A dose of podophyllin.

---

IN Bagdad, on May 6th, a 30-year-old Kurdish woman, the wife of a dealer in old clothes, died of a disease which showed all the symptoms of plague. All persons who had been in contact with the woman were placed under strict medical observation. By the orders of the supreme sanitary council in Constantinople, two temporary hospitals have been erected in Bagdad for the accommodation of those accompanying the passing caravans, and in which the travelers have to submit to five days' quarantine. The effects of the travelers are there subjected to a chemical disinfection and then daily exposed to the rays of the sun. Ships which have to leave Bagdad for the South have to undergo five days' quarantine in Gavarah. Cavalry patrols are to keep watch over the country between Bagdad and the hospitals, in order to turn back any fugitives. Official doctors and military physicians are to be sent into the neighboring villages of Bagdad in order to supervise the sanitary condition of the population. The duty of reporting every fresh case of plague will be strictly enforced.

---

A CHICAGO daily paper is authority for the following: Dr. Dudley S. Reynolds, one of the founders of the Louisville Hospital College of Medicine, and a member of its faculty from its inception, has been dismissed from the institution because of his antagonism to cigarette-smoking. He is now suing Central University, of which the medical school is a part, for $15,000 damages. Dr. Reynolds, in his lectures to his classes, denounced cigarette-smoking and smokers in unqualified terms. The students took offense and refused to attend his lectures unless an apology was made. This he declined to do. At this juncture the faculty joined with the students and requested his resignation. Again he declined, and his dismissal followed.

---

THE *New York Evening Post* is authority for the following: Recent inquiry at the offices of four of the largest hospitals in New York—namely, St. Luke's Episcopal, on Cathedral Heights; the Presbyterian, in Madison avenue; the German, at Park and Lexington avenues; and the Roosevelt, at Ninth avenue and Fifty-eighth street—brought out the fact that every one of them was struggling under a deficit, representing the difference between the institution's income and its expenditures for the year. In this respect the Presbyterian is perhaps the worst off, the treasurer reporting that during last year the current expenses exceeded receipts by $77,364.32. Next comes St. Luke's, with a balance of $62,043 on the wrong side; while the German and the Roosevelt are comrades in poverty to the extent of about $11,000 during the last year.

---

THE Sixteenth Annual Report of the Bureau of Animal Industry, Washington, D. C., contains an interesting account of the "Maladie du Coït" in horses, as observed in Nebraska. The infection in this case was traced in part to a stallion of the Clydesdale breed, which had been used for breeding purposes. A number of autopsies are reported in animals of both sexes which had been killed after the disease was discovered in them. In the male the affection is a local one, beginning in the penis, but involving sometimes the sheath, scrotum, and testicles. An edematous swelling extended in one case along the abdomen nearly to the forelimbs. An offensive purulent discharge occurs, and the entire end of the penis may slough away. The local sore is an ulcer, covered with a black scale. A skin eruption, consisting of large white maculæ, is observed. The animal emaciates and has to be killed. In the mare the infection may invade the whole genital tract and all the reproductive organs. In one case the white spots were seen on the perineum and mammary glands; the vagina was highly congested; the walls of the uterus were thickened, the inner surface being covered with a thick jelly-like exudate; and the ovaries were swollen to several times their normal size. These organs were the seats of extensive changes. There may be some systemic infection as shown in the mesenteric glands and spleen. This report contains no history of any bacteriological investigations, the accounts of the autopsies being confined to a description of the gross pathological conditions. The disease is evidently transmitted by coitus, and beginning as a local sore gradually extends, and may even invade the general system.

